# Integrated Design and Control of Various Hydrogen Production Flowsheet Configurations via Membrane Based Methane Steam Reforming

**DOI:** 10.3390/membranes9010014

**Published:** 2019-01-15

**Authors:** Alexios-Spyridon Kyriakides, Spyros Voutetakis, Simira Papadopoulou, Panos Seferlis

**Affiliations:** 1 Chemical Process and Energy Resources Institute (C.P.E.R.I.), Centre for Research and Technology Hellas (CE.R.T.H.), P.O. Box 60361, 57001 Thermi-Thessaloniki, Greece; alexkyr@cperi.certh.gr (A.-S.K.); paris@cperi.certh.gr (S.V.); 2 Department of Mechanical Engineering, Aristotle University of Thessaloniki, P.O. Box 484, 54124 Thessaloniki, Greece; 3 Department of Automation Engineering, Alexander Technological Educational Institute of Thessaloniki, P.O. Box 141, 57400 Thessaloniki, Greece; shmira@autom.teithe.gr

**Keywords:** hydrogen production, reactor modeling, dynamic process simulation, process optimization, low temperature methane steam reforming, multi-objective optimization, Integrated process design and control

## Abstract

This work focuses on the development and implementation of an integrated process design and control framework for a membrane-based hydrogen production system based on low temperature methane steam reforming. Several alternative flowsheet configurations consisted of either integrated membrane reactor modules or successive reactor and membrane separation modules are designed and assessed by considering economic and controller dynamic performance criteria simultaneously. The design problem is expressed as a non-linear dynamic optimization problem incorporating a nonlinear dynamic model for the process system and a linear model predictive controller aiming to maintain the process targets despite the effect of disturbances. The large dimensionality of the disturbance space is effectively addressed by focusing on disturbances along the direction that causes the maximum process variability revealed by the analysis of local sensitivity information for the process system. Design results from a multi-objective optimization study, where only the annualized equipment and operational costs are minimized, are used as reference case in order to evaluate the proposed design framework. Optimization results demonstrate the controller’s ability to track the imposed setpoint changes and alleviate the effects of multiple simultaneous disturbances. Also, significant economic improvements are observed by the implementation of the integrated design and control framework compared to the traditional design methodology, where process and controller design are performed sequentially.

## 1. Introduction

Chemical process design is a procedure that aims at determining the design point, both structural and operational, at which the process can fulfill the product specifications, sustainability requirements, and safety conditions while minimizing both the annualized equipment and operational cost. Process design procedure involves several steps [1]. After determining the input–output structure for the process system and subsequently the sub-systems structure, the synthesis of the process flowsheet through the selection of units performing specific tasks (e.g., reaction, separation, phase change) and their interconnectivity is then performed. The analysis step incorporates the determination of the variables that are related to the structural properties of the process (e.g., geometry and dimension, equipment type, and so forth) together with the calculation of the operating conditions to meet the production specifications within a predefined feasible range. Alternative design options are usually evaluated within an optimization framework, usually formulated as a non-linear mixed integer optimization problem [2], where the designer’s experience is exploited in order to reduce the design space. The problem is subject to several constraints imposed by the process physical model, described as a set of differential and algebraic equations and a set of inequality constraints. The equality constraints are used to describe the process under consideration (e.g., mass, energy and momentum balances), whereas inequality constraints are used to define the feasible space of the solution (e.g., operating limits). Decision variables can be both continuous (e.g., inlet flowrate) and integer, usually referring to structural decision variables (e.g., number of discrete reactor units). The objective function is usually related to the impact that the design variables have on process economics (e.g., capital and operational cost) but other criteria like safety, efficiency, environmental impact, waste generation, and so forth could also be considered. The problem of process design is based on steady state mathematical models that ignore the dynamics of the process and its variability when disturbance and uncertainties affect the process.

Nonetheless, under real operating conditions several factors influence the operation of the process system and cause a deviation from the optimal operating point. Such factors include disturbances, process inherent uncertainties and variations in the operating regimes. In order to meet process targets, a control system operates automatically, in order to compensate for the effect of disturbances. The achievable dynamic performance of the control system is dependent on several factors such as process design characteristics, operating conditions, the selected control algorithm and its tuning. The control design procedure involves the determination of the control objectives, input–output structure of the controller, controller algorithm selection and controller’s parameter tuning. The control objectives are defined as targets for measurable process variables closely related to the overall economic, sustainability, and safety specifications. As far as the input–output structure is concerned, several techniques [3] have been employed in order to define the number, location, and type of actuators and sensors used by a control system (e.g., minimum singular value, maximum singular value, condition number, relative gain array [4], and so forth). The tuning of the chosen control algorithm’s parameters is very much subject to the selected control algorithm. For example, well-established methods are present in the literature for the tuning of multi-loop PID controllers (e.g., [5,6]), whereas a range from offline methods to self-tuning (optimization-based) methods are available for the optimal settings of a model predictive controller (MPC) [7]. Moreover, in order to evaluate the candidate controller design, the system must be tested under the presence of anticipated disturbances under realistic plant operation.

The traditional approach is a successive procedure, where the calculation of the capacity related design parameters and the operating conditions is performed independent of the design of a suitable control system. However, the achievable dynamic and control system performance is limited primarily by the design decisions made in the process design step of this sequential approach. One approach to cope with the imposed limitations is to overdesign processes so that the effects of the expected disturbances and uncertainties are somewhat alleviated [8] usually leading to expensive process designs. Another approach for the attainment of the optimal dynamic performance of the system is to conduct process design procedure and control design procedure within a holistic, integrated framework. Both process and control system design aim at well related objectives focusing on steady-state and dynamic responses, respectively.

A large number of integrated process and control system design approaches are outlined by Seferlis and Georgiadis [9]. Similarly, Yuan et al. [10] presented an overview of the state-of-the-art and progress in optimization-based simultaneous design and control techniques. An attempt to classify the different methods based on the degree of integration was offered by Sharifzadeh [11]. On a similar base, Vega et al. [12] classified the available methods into projecting methods, where controllability criteria are utilized in trade-offs between design and control, and integrated-optimization methods, where several decomposition techniques are employed to handle the computational challenges of the problem.

Narraway et al. [13] and Narraway and Perkins [14] considered the economic penalty due to backing-off from active constraints during dynamic operation. The idea is based on the fact that steady-state designs are usually at the intersection of constraints and when the system undergoes dynamic transition due to a disturbance those constraints may become violated. The proposed framework calculated the necessary distance that the design variables must have from the active constraints so that the disturbances do not cause the system to violate those bounds assuming perfect control. The method was further extended to PI controllers in [15,16]. Further advancements on the back-off approach were presented by Mehta and Ricardez-Sandoval [17], where the cost function and the process constraints were represented by power series achieving a faster convergence to the optimal solution than previous works. A mixed integer dynamic optimization framework was presented by Mohideen et al. [18] where an iterative decomposition technique is utilized using multi-period problems for the characterization of uncertainty. Variability cost defined as possible economic losses induced by the dynamic performance was considered along with the annualized process capital and operating cost in [19]. Furthermore, the controller type selection under an integrated framework was analyzed for a system of CSTRs by Sanchez-Sanchez and Ricardez-Sandoval [20] and for a wastewater treatment plant by Gutierrez et al. [21].

Hydrogen is considered to be a promising energy carrier for the future. The increasing demand of hydrogen either as clean fuel alternative either as reactant in chemical industry needs to be addressed. Currently, the largest portion of hydrogen production is based on fossil fuels (mainly natural gas), while in smaller scales electrolysis is also utilized [22]. Regarding water splitting, research is mainly focused on the material over which the electro and photo-catalysis takes place in order to improve efficiency, durability, and cost. Examples of such works both experimental (e.g., [23]) and theoretical (e.g., [24,25]) can be found in literature. Referring to hydrogen production processes based on methane (natural gas) steam reforming, recent advances have resulted to the intensification of the process, where process integration and process design utilizing appropriate materials (e.g., catalysts, membranes) and equipment is gaining attention. Among other solutions, the utilization of a Pd-based membrane reactor is proposed. Such a reactor combines hydrogen production and hydrogen separation in a single step by exploiting the extremely high selectivity of Pd towards hydrogen. Hence, high methane conversion values at significantly lower operating temperature than in conventional reactors can be achieved [26]. As far as the design of such processes is concerned, limited articles deal simultaneously with the problem of process design and process control. A number of studies deals with the optimal design of membrane reactors both for the process of hydrogen production through methane steam reforming [27] as well as through ethanol steam reforming [28] by identifying the optimal operating conditions for the reactor system. Murmura et al. [29,30] employed a dimensionless analysis, where the influence of design parameters on the performance of the reactor was assessed and the operational and structural conditions over which reaction is truly active were identified. Moreover, Patrascu and Sheintuch [31] analyzed the process of the auto-thermal membrane reformer and defined the minimum reactor temperature that provides sufficiently high thermal efficiency. Regarding the dynamic behavior of reforming systems for hydrogen production, Silva [32] employed a one-dimensional, dynamic, isothermal model for the membrane reactor, whereas Ghouse and Adams [33] worked on a dynamic, two-dimensional, heterogeneous mathematical model. Wu et al. [34] developed a model predictive controller (MPC) which showed a superior closed loop performance than PI both for disturbance rejection and set-point tracking scenarios. Similarly, an optimal model predictive controller was also designed and implemented on an integrated membrane reactor system in [35].

Integrated process and control system design approaches have reached a certain degree of maturity in terms of method and employed techniques. Uncertainty considerations, advanced control schemes, and superstructure formulations enable the calculation of improved process design with enhanced dynamic characteristics. However, the implementation of such sophisticated techniques in highly intensified process model has not been thoroughly investigated [10]. As a result, the aims of this study it to further explore and advance the procedure of integrated process design and control for the optimal design of intensified process systems as the membrane-based hydrogen production via methane steam reforming using an advanced control system under multiple disturbance scenarios. A framework is presented, where both process and controller design are performed in an integrated fashion. Moreover, the proposed framework is applied for the integrated design and control of several alternative flowsheets for hydrogen production via methane steam reforming. Operational units, their interconnections, equipment sizing and the operating conditions are determined so that equipment and operational cost along with a dynamic performance index are minimized utilizing a predefined control algorithm under the presence of multiple disturbances affecting the system.

## 2. Integrated Process Design and Control

In the present work, the problem of the integrated design and control of processes is stated as a dynamic, nonlinear optimization problem, where the main goal is to define the design variables that minimize a cost function, representing the annualized investment and operational costs and dynamic performance criteria, related to closed-loop process performance.

### 2.1. Optimization Problem Formulation

This specific mathematical problem (Equation (1)) involves the minimization of the cost function *J* that depends on the state, output, and manipulated variables, (*x*(*t*), *z*(*t*), *u*(*t*)), continuous process design and controller tuning variables, (*d*, *d_c_*), integer variables associated to the process flowsheet configuration and to the control system input–output configuration, (*X*, *X_c_*), and variables associated to the realization of the disturbances and uncertainty parameters, (*w(t)*). The optimization problem is subject to the differential and algebraic equalities that describe the physicochemical process, (*f*, *h*), and the inequality constraints that define the process operating region, (*g*), both determining the feasibility space. Within this framework the control problem is embedded as an additional optimization problem. The corresponding controller performance index *J_MPC_* is subject to the differential and algebraic constraints of the controller problem (*φ*, *η*). The state, output, and manipulated variables of the process model employed by the controller are *χ*(*t*), *ζ*(*t*), and *y*(*t*), respectively.


$\underset{d,d_{c},X,X_{c}}{\mathrm{Min}}J\left(x\left(t\right),z\left(t\right),u\left(t\right),d,\;d_{c},X,X_{c},w\left(t\right)\right)$
(1)s.t.:
$f\left(\dot{x}\left(t\right),\;x\left(t\right),\;z\left(t\right),\;u\left(t\right),d,X,w\left(t\right)\right)=0$

h(x(t), z(t), u(t),d,X,w(t))=0

g(x(t), z(t), u(t),d,X,w(t))≤0

$\underset{u\left(t\right)}{\mathrm{Min}}J_{MPC}\left(\chi \left(t\right),\zeta \left(t\right),y\left(t\right),u\left(t\right),d_{c},X_{c},w\left(t\right)\right)$
s.t.:
$\varphi \left(\dot{\chi }\left(t\right),\;\chi \left(t\right),\;\zeta \left(t\right),\;y\left(t\right),\;u\left(t\right),d_{c},X_{c}\right)=0$

$\eta \left(\;\chi \left(t\right),\;\zeta \left(t\right),\;y\left(t\right),\;u\left(t\right),d_{c},X_{c}\right)\leq 0$


In its most general mathematical formulation (Equation (1)), the problem of integrated process design and control could also account for process structural alternatives reflected by different flowsheet configurations (*X*), different control system input–output configurations and controller types (*X_c_*). The design (decision) variables set (*d*, *d_c_*, *X*, *X_c_*) includes all variables related to the equipment cost (e.g., length, diameter, heat exchange area, and so forth) and the operating conditions (e.g., raw material feed rate, energy influxes, operating temperature/pressure, splitter ratio, and so forth). Suitable ranges are defined for all design variables.

An important aspect is the representation of process uncertainties and disturbances in the design optimization procedure. Historical process data can be utilized to identify disturbance scenarios that are expected to occur during the operation of the process and subsequently to influence the plant’s operating conditions. Each possible disturbance scenario involves multiple simultaneous disturbances of variable magnitude. It is critical to find an effective way to deal with multiple disturbances and uncertainties provided that this is a step that could severely alter the complexity of the procedure and intensify the computational demand. Within the proposed framework, a nonlinear sensitivity analysis is employed in order to effectively address the impact of disturbances on the control performance (Section 2.4).

### 2.2. Model Predictive Control

In the present work, a fully centralized controller is considered in the form of a linear multivariable model predictive control algorithm (MPC) [35] due to the highly interactive nature of the system under study. The MPC can optimally handle the interactions between the physicochemical phenomena occurring within the multivariable membrane reactor unit through an accurate dynamic model. Moreover, the explicit handling of constraints on both the manipulated and controlled variables enables the reactor operation close to constraints. Finally, the MPC can optimally handle time delays and inverse response, whereas optimal operation can be achieved when a surplus of manipulated variables is available.

### 2.3. Dynamic Performance Evaluation

In the present work, two indices are used for the evaluation of the dynamic performance of the process. The first index is described by Equation (2), and is utilized during the integrated process design and control of the alternative flowsheets for hydrogen production

(2) $$J_{MPC}=\sum_{i=1}^{N_{dyn}}J_{MPC,i}=\sum_{i=1}^{N_{dyn}}\left({\left(y_{sp}\left(i\right)-y\left(i\right)\right)}^{T}Q\left(y_{sp}\left(i\right)-y\left(i\right)\right)+\Delta u{\left(i-1\right)}^{T}R\Delta u\left(i-1\right)\right)$$

where *N_dyn_* is the number of discrete time intervals of dynamic simulation, *J_MPC,i_* is the value of the objective function of the MPC at time *i*, *y_sp_* is the set-point value at time *i*, *Δu* is the rate of change of the manipulated variables at time *i* and *Q* and *R* are weight matrices prioritizing the importance of the controlled output variables and the use of resources by the controller.

Even though index, *J_MPC_*, takes into account both the deviations from the desired set-point and the effort of the controller to minimize these deviations, it cannot be directly correlated to the economic effect. For this reason, a second index, *J_COST_*, linked directly to the economic losses that deviations from product specification during dynamic transition have is introduced. For example, if the production rate is considered as a controlled variable for which a specific target is set, then when that rate is higher than the target it means that unnecessary consumption of valuable raw materials occurs. On the contrary, when the production rate is lower than the target, valuable revenue is lost. The integral of the deviation between the actual production rate and the target level can be used to calculate the cost of dynamic transition and can be compared to equipment and operational costs. The annualized economic effect of the disturbance rejection is then multiplied by the expected frequency of occurrence for a specific disturbance scenario. The frequency of occurrence can be estimated based on process historical and experimental data for the nickel-based catalyst deactivation cycles and the membrane durability performance.

### 2.4. Direction of Maximum Variability

Considering the set of disturbances and uncertainties (*w*(*t*), of dimension *n_w_*) affecting the process under consideration, then a *n_w_*-dimensional disturbance space is formed. Each point in this high-dimensional space represents a different combination of disturbances and uncertainties at various magnitudes, whose realistic representation would require a sample consisted of a great number of points. Therefore, the computational effort to track the dynamic response over a representative number of disturbance scenarios would be extremely large.

Instead of investigating the behavior of the system at various disturbance scenarios, the disturbance scenario resulting in the worst-case scenario is considered (Ricardez-Sandoval et al. [19,36]). The worst-case scenario can be determined from the direction in the disturbance space that results in the maximum variability of the process variables. In order to define the direction over which the process variables are affected the most, a local sensitivity matrix, *P_w_* [37,38], needs to be obtained as described by Equation (3)

(3) $$P_{w}=\left[\nabla_{w}X\left(w_{nom}\right)\;\;\;\nabla_{w}\xi \left(w_{nom}\right)\right]$$

where *P_w_* is the local sensitivity matrix around a nominal operating point for the process, *X*(*w_nom_*) the process states calculated at the nominal values of disturbances and uncertainties and *ξ*(*w_nom_*) the eigenvalues of matrix *A* of the linearized, state-space model of the process calculated on the nominal values of disturbances and uncertainties. Term ∇*_i_x_j_* denotes the variation of *j*-th state (or *j*-th eigenvalue) when the *i*-th disturbance is perturbed around its nominal value (*w_nom_*(*i*)), as described by Equation (4)

(4) $$\nabla_{i}x_{j}=\frac{\partial x\left(j\right)}{\partial w\left(i\right)}=\frac{\left(x_{j}\left(w_{nom}\left(i\right)+\Delta w_{nom}\left(i\right)\right)-x_{j}\left(w_{nom}\left(i\right)-\Delta w_{nom}\left(i\right)\right)\right)}{2\cdot \Delta w_{nom}\left(i\right)}$$

Maximum variability for the process variables and eigenvalues is achieved for perturbations along the eigenvector of *P_w_^T^P_w_* that corresponds to the largest in magnitude eigenvalue. Information included in the sensitivity matrix are essential towards the definition of the direction of maximum variability because it contains both static (state) and dynamic (eigenvalue) information for the system. Other variables can be included in the sensitivity matrix in order to incorporate more information, for example generalized eigenvalues of matrix *A* where utilized by Seferlis [39] because they are related to transition zeros, which are responsible for inverse dynamic response.

### 2.5. Solution Procedure

**Figure 1:**
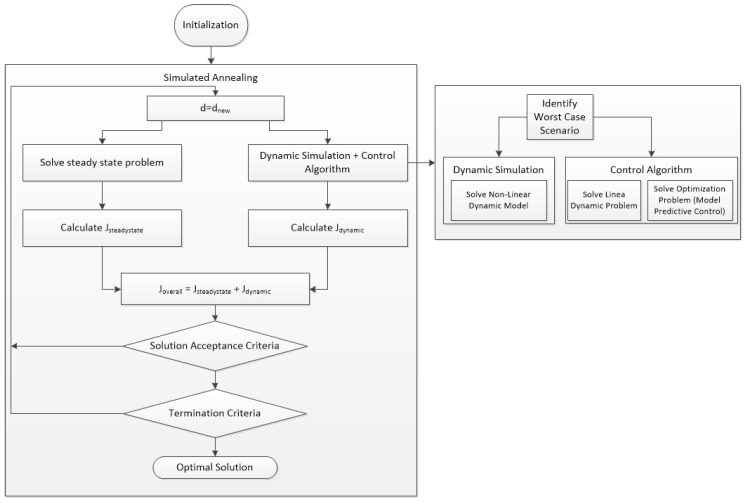
Solution procedure of the integrated process design problem with economic and control system performance criteria using simulated annealing.

The solution procedure for the non-linear dynamic optimization problem of Equation (1) is depicted in Figure 1. A simulated annealing algorithm, which is a probabilistic technique for approximating the global optimum of the objective function, is employed as it can deal with highly non-linear models and integer variables. However, the associated computational burden is a discouraging factor. Starting from an arbitrary point and then at every iteration of optimization algorithm a new set of values is assigned to the design variables (Figure 1, *d*). This set of values comprises each one of the design points that are going to be evaluated within the framework and is chosen by the simulated annealing algorithm within pre-defined limits. For each design point a steady state problem is solved, which is comprised by a set of non-linear algebraic equations (Figure 1, “Solve steady state problem”). The solution of this problem is used to calculate the annualized equipment and operating cost of the process, as well as to be used as a nominal operating point for the dynamic simulation of the process at each design point.

Successively, and for each one of the design points to be evaluated, the dynamic simulation of the process along with the solution of the control algorithm is performed, using the solution of the steady state problem as a starting point (Figure 1, “Dynamic simulation + control algorithm”). Given the controller type (MPC), and at every design point, the step of closed-loop simulation includes several tasks, the first of which is the linearization of the non-linear dynamic model which is utilized in the linear MPC. The second is the identification of the direction of maximum variability for the worst-case scenario. The next task is the dynamic simulation of the process (solution of the non-linear dynamic model) which is performed along with the solution of the controller algorithm (solution of linear dynamic model and the optimization of the MPC problem). Note that the optimization problem of the controller is solved at each time interval of the dynamic simulation, whereas the dynamic simulation is performed for each and every one of the design points evaluated by the framework. The dynamic simulation results are used to calculate the dynamic cost of the process.

Consecutively, the overall objective function (*J_OVERALL_*) is calculated utilizing properly adjusted weights multiplied to each term in order to reflect on their importance. Then if the algorithm’s specific solution acceptance criteria are met and if the algorithm’s specific termination criteria are met, the optimal solution is reached. During the optimization procedure, a candidate solution is always accepted if the value of the objective function is lower than the currently saved best solution. Additionally, if the value of the objective function is lower than the current solution, the new candidate solution is accepted randomly based on a probability depending on the difference between the objective functions value. Termination criteria refer to average change in objective function value, maximum number of iterations or maximum simulation time. Given the complexity of the framework and the process, the whole procedure is repeated for several times starting from a new, random within pre-defined bounds, arbitrary point and until the value of the objective function is not improving any further.

MATLAB software was the basis for the development and implementation of the presented framework [40]. Simulated Annealing algorithm was used for the optimization problem, while a quadratic programming technique was implemented for the control problem in order to determine the optimal control actions at each interval in the closed loop simulation. An ordinary differential equation solver (ode15s) of MATLAB was used for the dynamic simulation.

## 3. Results

### 3.1. Case Study: Hydrogen Production via Methane Steam Reforming

**Figure 2:**
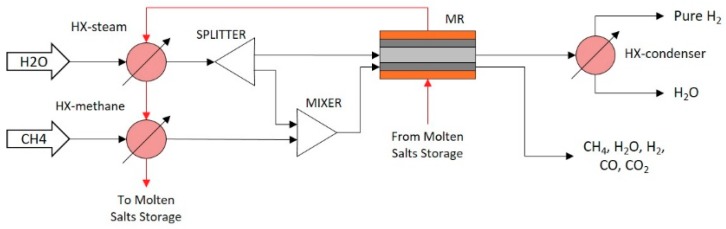
Integrated membrane reactor flowsheet configuration (IMR).

**Figure 3:**
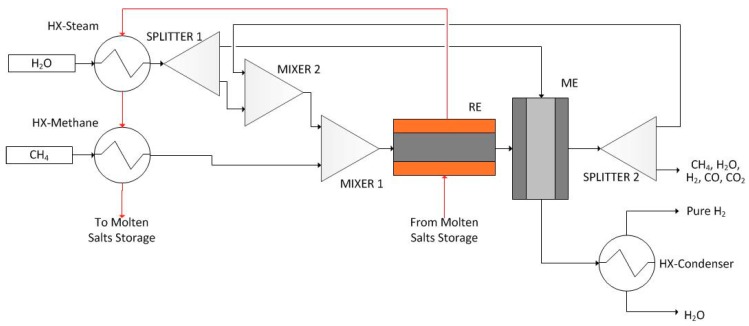
Cascaded reactor-membrane flowsheet configuration (CRM).

**Figure 4:**
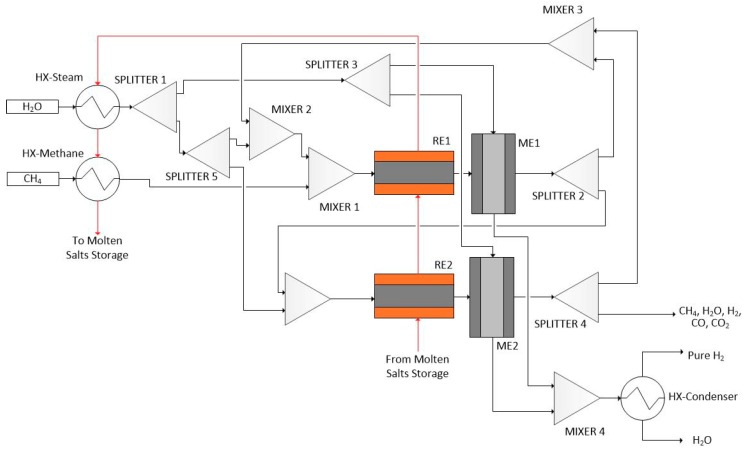
Cascaded multiple reactor-membrane flowsheet configuration (CRMRM).

Hydrogen production through methane steam reforming at low temperature is optimally designed using the proposed integrated framework. Methane and water are fed into the reactor. The reactions take place over a Ni-based catalyst supported by foam (SiC with porosity of 85%). Operating reaction temperature is in the range of 723–823 K whereas pressure is held at 10^6^ Pa. Hydrogen, carbon monoxide, and carbon dioxide are the products. Additionally, hydrogen is separated either in an integrated Pd-based membrane reactor module (Figure 2), or in a Pd-based membrane separation module (Figure 3 and Figure 4). Either a single reactor (Figure 2 and Figure 3) or multiple reactors (Figure 4) can be employed. The mathematical model of the reactor has to take under consideration the complex reaction mechanism of the reforming reaction, the convective and molecular diffusion of the reacting mixture species and the thermal effects due to the endothermic reactions and the heat exchange with an external source. The reaction scheme and kinetic models of the aforementioned process are described in detail by Kyriakides et al. [26].

#### 3.1.1. Alternative Process Flowsheets

The developed framework is utilized for the optimal design and control of three alternative flowsheet configurations. The first flowsheet is equipped with an integrated membrane reactor. The other two configurations utilize traditional tubular reactor modules followed by a membrane-based separation module, also exploiting the advantages of hydrogen dilution in palladium. The succession of individual reactive and separation modules offers effective protection of the membrane against the conditions prevailing in the reactor that may have an impact on the membrane lifetime [41]. On the contrary, the thermodynamic limitation on the achieved methane conversion for the successive production and separation configuration is the key disadvantage.

##### Integrated Membrane Reactor Configuration (IMR)

The integrated membrane reactor flowsheet configuration (IMR) shown in Figure 2 is consisted of an integrated membrane reactor module and three heat exchangers. Methane and steam are heated up to the reaction temperature utilizing two heat exchanger modules (HX-Steam and HX-Methane). Steam flowrate is divided in a splitter (SPLITTER) so that one stream is mixed with methane in a mixer module (MIXER) providing the reacting mixture and the second is used as sweep gas in the membrane reactor (MR) for hydrogen separation driving force maximization. The experimental unit on which the membrane reactor is based on is described in detail by Kyriakides et al. [26]. The geometry of the reactor consisted of three radial zones, namely the reaction, permeation and the molten salts zones and the operational constraints can be found in Kyriakides et al. [42]. The molten salt loop that is utilized in order to provide the necessary heat to the membrane reactor is consecutively used to preheat the reactants in the steamer and the heat exchanger modules. The membrane is a 4–5 μm Pd-based, selective layer which is coated on a ceramic dense support (porous Al_2_O_3_). The driving force for hydrogen removal through the membrane is proportional to the difference of the square root of hydrogen partial pressure between the reaction and the permeation zone. Finally, the permeate stream is consecutively fed to a condenser (HX-condenser) where steam is condensed and a pure hydrogen stream is obtained.

##### Cascaded Reactor and Membrane Modules (CRM)

The cascaded reactor and membrane flowsheet configuration (CRM) is shown in Figure 3. Comparing to the IMR flowsheet, a reactor module and a membrane-based separation module are connected in series. In a similar fashion, methane and water are heated up to the reaction temperature utilizing two heat exchanger modules (HX-steam and HX-methane). Consecutively, steam stream is divided in a splitter (SPLITTER), where the first of the two streams is used as sweep gas stream in the membrane-based separation module (ME). The second stream is mixed in a mixer module (MIXER 2) with the recycled stream exiting the membrane module, which contains unreacted methane and steam along with carbon monoxide and carbon dioxide produced in the reactor module and hydrogen that was not separated in the membrane module. The resulting stream is then mixed in a mixer (MIXER 1) with methane feed, providing the reacting mixture that is fed to the reactor module (RE). Reaction conditions are similar to those in the IMR flowsheet configuration, but the reactor is consisted of two concentric tubes instead of three (reaction and molten salt zones). At the reactor outlet, the mixture is at equilibrium composition and is fed to the separator (ME), of similar geometry as the RE module (permeation and reaction retentate zone) in order for hydrogen to be separated. The second stream exiting the separator module (ME) containing the sweep gas as well as the separated hydrogen is fed to a condenser (HX-condenser) for hydrogen purification.

##### Cascaded Multiple Reactor and Membrane Modules (CRMRM)

The third investigated flowsheet (CRMRM) is shown in Figure 4. The process is similar to the CRM flowsheet (Figure 3) but with two successive reactor and membrane-based separation modules. In this way reactor RE1 outlet stream, which is a mixture that is at equilibrium conditions, is fed to separator ME1 in order for hydrogen to be removed. Consecutively, separator ME1 outlet mixture, containing the remaining unreacted steam, methane and carbon monoxide and carbon dioxide produced as well as remaining, unseparated hydrogen, is fed to a mixer (MIXER 5). The mixture is then enriched in terms of steam so that the steam to carbon ratio at the inlet of reactor RE2 is in agreement with process operational specifications. Steam to carbon values of less than two favor coke formation, which results in catalyst deactivation and should be avoided. Outlet stream of both separator ME1 and separator ME2 containing sweep gas and separated hydrogen are mixed in mixer (MIXER 4) and then fed to a condenser (HX-condenser) for pure hydrogen separation. Additional splitter and mixer modules are utilized so that streams from both separator modules can be recycled and re-used in the first reactor (RE1).

#### 3.1.2. Mathematical Modeling of the Alternative Flowsheets

Physicochemical phenomena occurring inside the modules that comprise the flowsheets under consideration are described by dynamic mathematical models based on Kyriakides et al. [35] and [42]. The dynamic simulation involves the solution of a set of partial differential equation, consisted of the dynamic mass and energy balances of the reaction and separation modules in temporal and spatial dimension as well as the dynamic mass and energy balances of the additional modules. The partial differential equations are discretized in the axial direction utilizing the finite difference scheme. A dynamic, nonlinear, one-dimensional, pseudo-homogeneous mathematical model of the multi-tubular, membrane reactor is employed. The model comprises: (a) mass balances for every component (*i* = CH_4_, H_2_O, H_2_, CO, CO_2_), Equation (5), and (b) energy balances, Equation (6), in each one of the three zones.

(5) $$\frac{\partial C_{i}}{\partial t}=-u\frac{\partial C_{i}}{\partial z}+a\rho_{b}\overset{3}{\underset{j=1}{\sum }}R_{j}\nu_{i,j}-b\frac{2r_{i}}{r_{o}^{2}-r_{i}^{2}}N_{m}+c\frac{2r_{i}}{r_{i}^{2}}N_{m},\;i={\mathrm{CH}}_{4},H_{2}O,H_{2},\mathrm{CO},{\mathrm{CO}}_{2}$$

(6) $$\rho C_{p}\frac{\partial T}{\partial t}=-u\rho C_{p}\frac{\partial T}{\partial z}+d\rho_{b}\overset{3}{\underset{j=1}{\sum }}\Delta Hr_{j}R_{j}+d\frac{2r_{o}}{r_{o}^{2}-r_{i}^{2}}h_{w}\left(T_{ms}-T\right)+e\frac{2r_{o}}{r_{ms}^{2}-r_{o}^{2}}h_{w}\left(T_{r}-T\right)$$

Binary parameters *a*, *b*, *c*, *d*, and *e* are used in order to match the equations to the corresponding phenomena that take place at every zone of the membrane reactor. Parameter *a* is equal to unity when the equation refers to a reaction zone for every component, whereas is equal to zero for any other zone. Parameter *b* is equal to unity when referring to hydrogen in reaction zone. Parameter *c* is equal to unity when applied to hydrogen in the permeation zone and equal to zero otherwise. Parameter *d* is equal to unity when applied to the reaction zone and equal to zero otherwise, and finally, parameter *e* is equal to unity when referring to molten salt zone and equal to zero otherwise. The same mathematical model is adapted for the CRM and CRMRM flowsheet configurations.

#### 3.1.3. Optimal Design of Process Flowsheet

At first, the traditional procedure of the optimal design of the alternative flowsheet configurations based on steady state mathematical models is performed. This step is critical in order to obtain a basis over which the capabilities of the proposed integrated process design and control framework can be compared to. The objective of the traditional optimal design procedure is the minimization of the equipment and the operational cost of the process. Total capital cost is expressed as the sum of the cost of the membrane reactor (IMR) or the cost of the reactor and the membrane (CRM and CRMRM) and the cost of the heat exchanger involved in each configuration, as described by Equation (7)

(7) $$J_{1}=CAPEX=C_{MR}+\overset{N_{RE}}{\underset{i=1}{\sum }}C_{RE}+\overset{N_{ME}}{\underset{i=1}{\sum }}C_{ME}+\overset{N_{HX}}{\underset{i=1}{\sum }}C_{HX}$$

The total operational cost is expressed as the sum of the cost of methane and water as well as the cost of the cooling water utilized in the condensers at each configuration and is described by Equation (8)

(8) $$J_{2}=OPEX=C_{{\mathrm{CH}}_{4}}+C_{H_{2}O}+C_{\mathrm{water}}$$

The membrane reactor cost is evaluated based on the relations employed by Marechal et al. [43] and Sanusi et al. [44] whereas heat exchanger cost is based on Walas [45]. Additionally, the annualized capital cost is evaluated based on the cash recovery factor (CRF). The annualized operational cost is based on the unit cost of each parameter (methane, water, cooling water) multiplied by its flow rate and the expected number of operation hours, chosen to be equal to 8000 h.

A multi-objective design optimization procedure is employed for the optimal design of all alternative flowsheet configurations. The resulting Pareto front enables the investigation of the trade-offs between the competing objective functions.

##### Design Optimization Problem Formulation

The general mathematical formulation for the multi-objective optimization problem is presented in Equation (9). A number (*k* ≥ 2) of objective functions *J_i_*(*x*) are to be simultaneously optimized. The minimization problem is subject to *m* equality *f*(*x*) and *q* inequality constraints *g*(*x*) that define the feasible space of the solution. The objective functions, equality constraints and inequality constraints are functions of the design variable vector of length *n*.

(9) $$\begin{array}{ccc} & \;\underset{x}{\mathrm{Min}}\left(J_{1}\left(x\right),\;J_{2}\left(x\right)\ldots J_{k}\left(x\right)\right) & \\ s.t.: & f\left(x\right)=0,\;g\left(x\right)\leq 0 & x\in R^{n},\;f\left(x\right)\in R^{m},\;g\left(x\right)\in R^{q} \end{array}$$

Through the multi-objective optimization, a set of compromised solutions are obtained. These solutions are called non-dominated or Pareto front if none of the objectives can be improved without decreasing at least another objective of the optimization problem. In order to obtain the set of solutions a controlled, elitist genetic algorithm (a variant of NSGA-II), is employed in order to solve the multi-objective optimization problem [46].

The structural decision variables for the IMR configuration are the membrane diameter (*R_i_*), the reactor’s outer diameter (*R_o_*), the molten salt’s zone outer diameter (*R_ms_*), the membrane reactor’s length (*L*), and the heat exchangers’ area. The operational decision variables that are optimized are the water and methane inlet flow rates (*F*_H2O_ and *F*_CH4_, respectively) as well as the splitter ratio at the SPLITTER module. The structural decision variables for the CRM configuration are the reactor’s geometry (defined by inner and outer diameters, and length) and separation module’s geometry (defined by inner and outer diameters and length, as well) and the heat exchangers’ area. The operational decision variables are the water and methane inlet flow rate and the splitter ratio at both splitter modules. For the (CRMRM) configuration the structural decision variables are both reactors’ geometry, both separation modules’ geometry and the heat exchangers’ area. The optimized operational variables are water and methane inlet flow rates and splitter ratio at each splitter module. All of the decision variables are chosen to vary within predefined physical limits, as shown in Table 1. In addition, steam to carbon ratio is constrained between two, to avoid coke formation, and four. Pure hydrogen production rate is constrained between 1.58 × 10^−5^ Nm^3^/s and 1.75 × 10^−5^ Nm^3^/s.

##### Design Optimization Results

**Figure 5:**
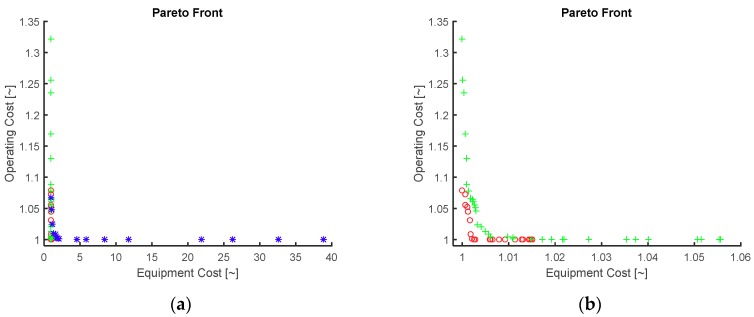
Pareto optimal solutions for the simultaneous minimization of equipment and annualized cost of (**a**) all three alternative configurations and (**b**) the IMR and CRM configurations. IMR configuration (red circle), CRM (green plus) and CRMRM (blue asterisk).

The Pareto optimal solutions of the three alternative configurations are shown in Figure 5. Both equipment and operational cost are scaled with respect to each configuration’s lowest value for each objective. Results indicate that the fluctuation of equipment cost for the IMR and CRM flowsheets is much lower (less than 2% and 6%, respectively) compared to the costs in the CRMRM flowsheet (up to 40 times). This behavior is attributed to the much larger equipment capacity as well as to the additional modules required by the CRMRM configuration. On the other hand, the operating cost fluctuates up to 35% on the solutions on Pareto front.

However, in all cases the single optimal solution is also obtained and presented in Table 2, Table 3 and Table 4 (OD column). In order to obtain a single best solution of the Pareto front a global criterion decision making method is utilized, where the distance of every solution on the Pareto front from the ideal solution is the criterion. This single optimal solution obtained within this section is used as a reference case for comparison with the design results from the integrated design and control procedure. The LINMAP method [47] is used to select a single design point from the Pareto front as described in Equation (10)

(10) $$\min\left(\overset{n}{\underset{i=1}{\sum }}d_{i}=\overset{n}{\underset{i=1}{\sum }}\overset{m}{\underset{j=1}{\sum }}{\left(F_{i,j}-F_{j,ideal}\right)}^{2}\right)$$

where *m* is the number of objective function and *n* is the number of optimal solutions on the Pareto front. The solution that has the minimum index *d* is the solution, which has the minimum distance from the ideal solution and is considered the single desired optimal solution.

Based on the optimal results IMR configuration utilizes the least quantities of reactants, whereas the CRMRM configuration utilizes tenfold higher resources. The heat exchanger areas are significantly bigger in CRM and CRMRM than in IMR configuration, whereas the membrane reactor in the IMR configuration is more compact than in the other two configurations. Additionally, the CRMRM configuration requires much larger reactor diameters than the other configurations due to much higher recycle streams. Finally, the membrane module in CRM utilizes a lower membrane surface compared to both other alternative configurations. In Table 2 the values of equipment and operational cost of all alternative flowsheets are normalized based on the values obtained for the IMR configuration. Both IMR and CRM exhibit similar equipment costs, whereas the CRMRM alternative is more expensive by a factor of three. The small difference in equipment cost values between the IMR and CRM configurations is attributed to the small difference in the membrane area (lower in CRM) and the extra operational unit in CRM configuration. Regarding operational cost, CRM configuration results in three times higher cost than IMR, whereas CRMRM configuration requires up to 10 times higher cost than IMR. The difference is attributed to thermodynamic limitations in the cascaded flowsheets that require significantly higher methane inlet flowrate in order to produce the same amount of pure hydrogen.

#### 3.1.4. Integrated Design and Control Framework Results

The steady state design problem cannot address the ability of the process system to operate under variations imposed by exogeneous disturbances and uncertainty associated with the process model. Frequently, the steady state optimal point is overdesigned by arbitrary factor to account for such variability. The proposed integrated design and control framework aims to address the design problem in a systematic way. In addition to the process design the control problem must be defined.

A control problem is defined for the integrated design and control framework that aims to track a 15% set-point increase in pure hydrogen production flowrate (*F*_H2*,out,p*_, controlled variable) with a simultaneous multiple disturbance rejection scenario, towards the direction of maximum variability. The possible disturbances are related to variations in the Sieverts law pre-exponential coefficient (*Q_mem_*) to emulate potential membrane deactivation, the reaction coefficient (*ReaCoef*) to emulate potential catalyst deactivation, and the molten salt inlet temperature (*T_ms,in_*) to emulate solar trough efficiency fluctuations. As manipulated variables, water and methane inlet flowrate (*F*_H2O_) and (*F*_CH4_) and splitter ratios (SP1Ra, SP2Ra, SP3Ra, SP4Ra and SP5Ra, where applicable) are selected.

The employed model predictive controller is tuned for the steady state optimal design point shown in Table 3 and Table 4. The objective function for the IDC problem is shown in Equation (11)

(11) $$\underset{d}{\mathrm{Min}}J=\left(\begin{array}{c} Equipment\;Cost\;\left(J_{1}\right)\\ +Operating\;Cost\;\left(J_{2}\right)\\ +Dynamic\;Performance\;\left(J_{COST}\;or\;J_{MPC}\right) \end{array}\right)$$

where the equipment and operational cost are calculated based on Equations (7) and (8). Two alternative indices, namely *J_COST_* and *J_MPC_*, are used in order to calculate the dynamic performance. Each term of the objective function is multiplied by properly adjusted weighting constants, chosen by the designer in order to depict on the importance of each individual term.

Comparative results regarding the multi-objective steady-state design optimization results (denoted as OD) and the integrated process and control system design (denoted as IDC) using two different indices for the evaluation of the dynamic performance (*J_COST_* and *J_MPC_*) are presented in Table 3 (IMR) and Table 4 (CRM and CRMRM). IDC framework must be capable of accounting for the effect of disturbances and therefore the overdesign is optimally calculated. As a result, by observing the optimization results equipment and operating cost tend to be lower for the OD design procedure compared to the IDC framework for all flowsheet configurations. Methane inlet flowrate utilization, which is the decisive factor of the operating cost level, is lower for all configurations. Water inlet flowrate utilization is higher for IMR and CRMRM configurations and similar for the CRM configuration. Heat exchanger areas calculated by IDC are either similar in the case of CRMRM or larger in the case of IMR and CRM configurations compared to the values calculated by OD. However, the membrane reactor (IMR) has a similar inner diameter in both design procedures. In addition, IDC procedure obtains a higher outer and molten salt diameters and reactor length than OD for IMR configuration. As a result, lower equipment cost is obtained by the OD procedure compared to the IDC framework. This is expected as OD procedure focused only on the minimization of the steady state costs. Additionally, both reactor and separation units’ size are lower compared to the IDC results for CRM and CRMRM configurations. However, regarding the CRMRM configuration designed by IDC procedure, lower membrane surface is used in the first separator and higher in the second separator unit. This result is attributed to the fact that steam to carbon ratio limits are less strict for the second reactor operating unit. Higher steam to carbon ration results to higher methane conversion and larger quantity of hydrogen to be separated in the second separation unit. Such behavior is due to the fact that reactive mixture reaches equilibrium state at the first reactor unit. Subsequently, although hydrogen is removed from the mixture (first separator), its concentration (given similar operating pressure and temperature) is much closer to the thermodynamic equilibrium conditions, limiting the achievable methane conversion in the second reactor unit. By employing a higher steam to carbon ratio, the equilibrium is shifted towards hydrogen production. On the down side, a significantly larger total water inlet flowrate is utilized in this flowsheet configuration.

Comparative results regarding the application of the IDC and OD framework, on all the flowsheets are presented in Table 5. Equipment, operating, and dynamic performance costs are normalized based on the performance of the IMR flowsheet values optimized within the OD procedure. Thermodynamic limitations due to low operating temperature seem to affect the cascaded configurations more, especially the CRMRM, than the IMR configuration. In order to produce the same amount of pure hydrogen, larger inlet flowrates are required resulting to bigger operating units as well. As a result, operational cost is higher than in IMR flowsheet, whereas equipment cost is similar between IMR and CRM but much higher in CRMRM. The significant difference is attributed to the additional reactor and separation operational units.

Both equipment and operational cost are higher compared to the OD procedure. For example, in the IMR case the proposed framework employing the *J_COST_* index results to 7% higher equipment cost and 21% higher operational cost compared to the OD case, where the dynamic performance of the process is not considered. Additionally, comparison of the *J_COST_* and *J_MPC_* indices, shows a slightly higher equipment and operational cost when *J_MPC_* is employed. Similarly, in the CRM configuration, all design procedures result to higher equipment cost compared to the IMR configuration. Referring to the CRMRM configuration, in all cases both operational and equipment costs are significantly higher, revealing the economic inadequacy compared to the alternatives evaluated within the present work. Moreover, the utilization of both indexes results to the same behavior, where the *J_MPC_* results to slightly higher equipment and operational cost.

However, by comparing the dynamic operation cost, calculated by evaluating the economic effect of the off-spec performance, IDC framework presents superior performance compared to the OD procedure for all alternative flowsheets. For example, and as far as the IMR configuration is considered, the IDC design results to 17% and 16% lower dynamic operational cost, using *J_COST_* and *J_MPC_* index, respectively. Another useful observation is the fact that both cascaded configurations present significantly lower dynamic operational cost (OD procedure), compared to the IMR (from 0.83 to 0.63). Similarly, CRM and CRMRM configurations perform even better when designed using the IDC framework (on average 48% and 54% lower dynamic cost compared to the OD of the IMR configuration). Additionally, by comparing the employment of the two indices, similar improvement on dynamic operating cost is observed. For example, IMR configuration designed using IDC performs 17% and 16% better than the OD using *J_COST_* and *J_MPC_* index, respectively. However, the improvement in the dynamic performance for the CRM and CRMRM is achieved at the expense of extremely higher equipment and operational costs than the IMR case. The higher available capacity provides to the system the necessary resources to effectively compensate for the disturbances. In particular, the equipment cost in the CRMRM configuration is three times higher and the operating costs 10 times higher than the IMR. Similarly, for the CRM configuration the equipment cost is 3% higher and the operating costs is three times higher than the IMR.

**Figure 6:**
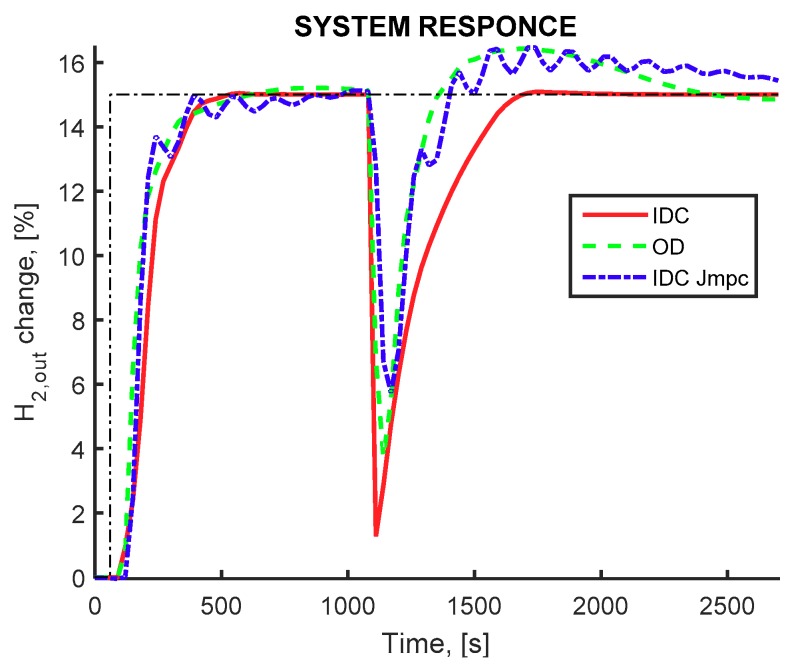
Pure hydrogen production rate (controlled variable). IMR flowsheet equipped with a membrane reactor, IDC(*J_COST_*) framework (red continuous curve) OD framework (green dashed curve) IDC(*J_MPC_*) framework (blue dashed-dot curve). Setpoint change for hydrogen production at 150 s and disturbance influence at 1000 s.

The dynamic behavior of the closed loop simulation of the IMR configuration display the ability of the controller to compensate for the tracking of the setpoints and for the disturbance rejection, and is shown in Figure 6. The controlled variable mainly deviates from the desired setpoint value at the time instance where the changes in the membrane permeability, catalyst activity and molten salt inlet temperature are imposed. In all flowsheet configurations, the recovery of hydrogen production rate is achieved with adjustment of the methane and water inlet flowrates as well as the split ratio(s). Additionally, the IDC using the cost related index (red curve) results in slightly overdamped slower responses with small overshoot. The multi-objective design (green curve) results to smaller rise time but larger settling time, similar to the IDC using the MPC related index (blue curve). The ability of the proposed framework to provide a better, in terms of dynamic response behavior, is therefore illustrated.

**Figure 7:**
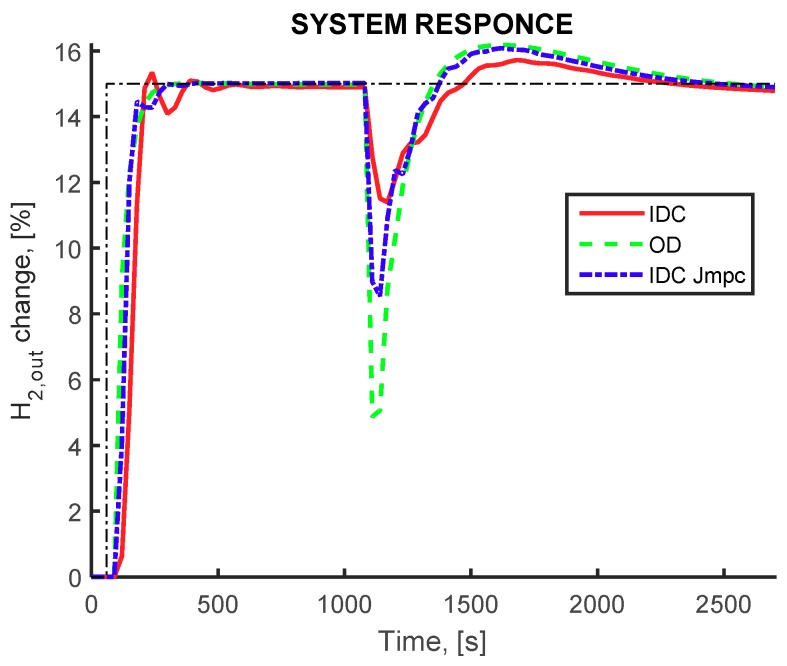
Pure hydrogen production rate (controlled variable). CRM flowsheet, IDC(*J_COST_*) framework (red continuous curve) OD framework (green dashed curve) IDC(*J_MPC_*) framework (blue dashed-dot curve). Setpoint change for hydrogen production at 150 s and disturbance influence at 1000 s.

Regarding the CRM flowsheet, the closed loop simulation under the same set-point tracking and worst-case disturbance scenario is presented in Figure 7. All three alternative designs perform very good under the set-point tracking scenario (at time *t* = 150 s), whereas at the time instances that the worst-case multiple disturbance scenario is imposed at the system (at time *t* = 600 s), the IDC framework using *J_COST_* index (red curve) show better behavior compared to the other two cases, where the response display smaller deviation from the desired set point level. Additionally, the IDC framework using *J_MPC_* index (blue curve), although slightly worse than *J_COST_* index, presents a better dynamic performance compared to the OD results, especially during the disturbance rejection task. The ability of the proposed framework to provide better results, in terms of dynamic response behavior, is illustrated for both tested indexes.

**Figure 8:**
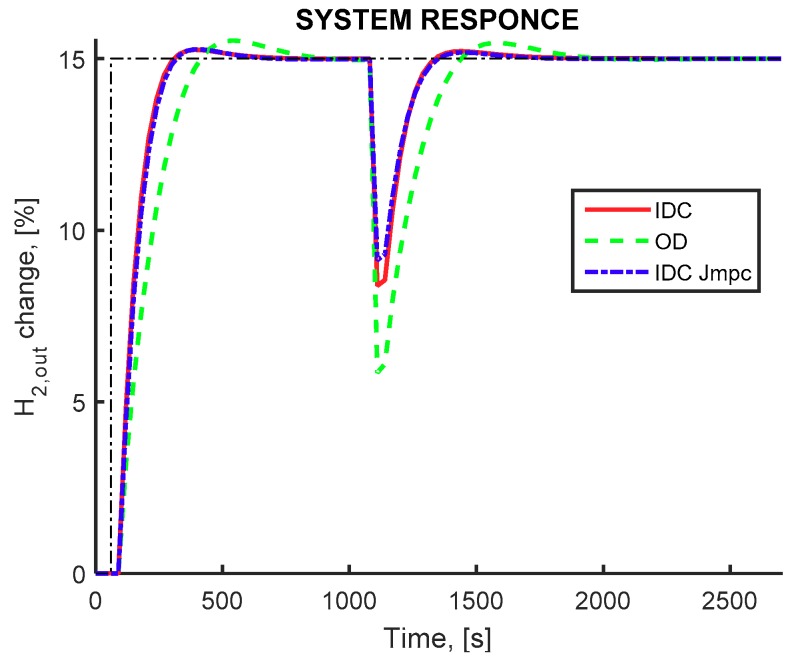
Pure hydrogen production rate (controlled variable). CRMRM flowsheet, IDC(*J_COST_*) framework (red continuous line) OD framework (green dashed line) IDC(*J_MPC_*) framework (blue dashed-dot line). Setpoint change for hydrogen production at 150 s and disturbance influence at 1000 s.

The closed loop simulation of the optimally designed CRMRM configuration when the worst-case disturbance scenario is imposed to the system is presented in Figure 8. In all three cases the controller is able to counteract the effect of the disturbance scenario. Additionally, the IDC framework was able to produce significantly better flowsheet designs, in terms of dynamic performance. Both of the two employed indexes display faster response during track point change compared to the multi-objective framework results. Moreover, during the disturbance rejection IDC designs show smaller deviation from the set point.

**Figure 9:**
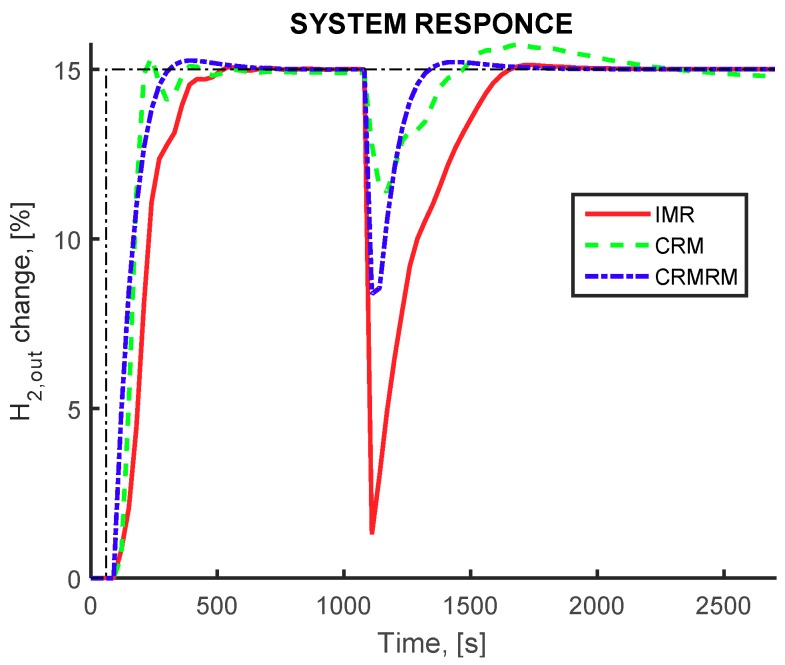
Pure hydrogen production rate (controlled variable). IMR (red continuous curve), CRM (green dashed curve), and CRMRM flowsheet (blue dashed-dot curve). Setpoint change for hydrogen production at 150 s and disturbance influence at 1000 s.

Finally, the performance of all three alternative flowsheet configurations using the dynamic cost related index in the objective function, are presented in Figure 9. By comparing the response of pure hydrogen production during the setpoint tracking and the disturbance rejection the superior dynamic performance of the cascaded reactor–separator configuration can be observed. Both cascaded configurations have faster response and can cope with the effect of the multiple disturbances displaying smaller deviation compared to the IMR configuration. This behavior is attributed to the disengagement of the physicochemical phenomena occurring in the reactor and the separator modules compared to the intensified membrane reactor unit, as well as to the extra manipulated variables that these configurations employ with the additional splitters and the recycle streams. In addition, the larger reactor and membrane separator modules in the cascaded configuration compared to the IMR configuration allow for a more efficient attenuation of the disturbances and subsequently assisting the controller to achieve a better performance. Of course, the superior dynamic performance is achieved at the expense of much higher equipment and operational costs. Considering the overall performance in steady and dynamic state the IMR provides the best design option as it offers a trade-off between the cost forms.

## 4. Conclusions

A systematic and comparative assessment of a number of alternative flowsheet design configurations of hydrogen production processes utilizing a Pd-based membrane is performed. A methodology for the holistic optimal process design and control of chemical processes is developed and further utilized in the optimal design of a number of alternative process configurations. The present framework takes into account the complete dynamic performance of the system under closed loop conditions with the implementation of an advanced optimal control scheme. Moreover, the current holistic process design and control framework is applied to a challenging and highly intensified and complex process compared to processes considered in previous works. Finally, the proposed design framework is unique in combining the design and advanced control of highly intensified processes with a stochastic optimization algorithm that ensures the identification of the global optimal solution under multiple simultaneous disturbances.

The simultaneous minimization of the annualized equipment and operational cost reveal the economic superiority of the IMR configuration against the cascaded flowsheets. The holistic optimization study was able to ensure efficient operation under steady state and dynamic transitions. Even though, equipment and operating costs are lower for the OD procedure, the off-spec and dynamic performance is better in the holistic design approach. For example, regarding the holistic design of the IMR configuration, equipment cost is on average 8% higher and the operating cost is on average 22% higher but the dynamic performance results to a better economic performance by 26.5%. Similarly, CRM and CRMRM flowsheets, when designed through the IDC framework, perform significantly better in terms of dynamic performance, compared to OD procedure. IMR enables the most economic design with respect to equipment and operating cost with acceptable dynamic performance over CRM and CRMRM configurations. The holistic approach optimally overdesigns the alternative process flowsheets through a systematic process so that the most effective process system is achieved under real operating conditions, where process variability is a key factor that deteriorates economic performance. The optimal distribution of resources expressed in terms of either additional equipment capacity or process streams is dictated by the optimal alleviation of the process inherent and other exogenous sources of variation from the control and process objectives. As a result, minimal increase in equipment and operating cost is achieved for a substantial degree of improvement in dynamic performance in all cases. The holistic design approach considering the closed loop dynamics offers competitive solutions for highly intensified process systems such as the integrated membrane reactor for methane steam reforming that would otherwise be extremely difficult to design and control. Finally, the proposed design approach is systematic, rigorous, and generic in nature and can therefore be adopted for applications of similar complexity.

## Figures and Tables

**Table 1:** Decision variable ranges employed in the optimization of the alternative configurations

Decision Variable	Limits					
	IMR		CRM		CRMRM	
	Lower	Upper	Lower	Upper	Lower	Upper
Membrane diameter (m)	10^−4^	0.1	-	-	-	-
Reactor outer diameter (m)	10^−4^	0.1	-	-	-	-
Molten Salt outer diameter (m)	10^−4^	0.1	-	-	-	-
Reactor *i* diameter (m)	-	-	10^−4^	0.1	10^−4^	0.2
Molten salt *i* outer diameter (m)	-	-	10^−4^	0.2	10^−4^	0.5
Membrane *i* diameter (m)	-	-	10^−4^	0.1	10^−4^	0.2
Separator *i* outer diameter (m)	-	-	10^−4^	0.2	10^−4^	0.5
Reactor *i* length (m)	10^−2^	1.0	10^−2^	1.0	10^−2^	5.0
Separator length (m)	-	-	10^−2^	1.0	10^−2^	5.0
Water inlet flowrate (×10^−5^ m^3^/s)	0.0224	2.24	0.0224	22.4	0.0224	560
Methane inlet flowrate (×10^−5^ m^3^/s)	0.0224	2.24	0.0224	22.4	0.0224	224
Splitter *i* ratio (-)	0.1	0.9	0.1	0.9	0.1	0.99
Steamer heat exchanger area (m^2^)	0.01	1.0	0.01	1.0	0.01	1.0
Heat exchanger area (m^2^)	0.01	1.0	0.01	1.0	0.01	1.0
Condenser heat exchanger area (m^2^)	0.01	1.0	0.01	1.0	0.01	1.0

**Table 2:** Equipment and operational cost on the single optimal solution for each configuration

Cost	IMR	CRM	CRMRM
Equipment	1.0	1.03	2.96
Operational	1.0	3.10	10.64

**Table 3:** Decision variables for the IMR flowsheet. Comparison between optimal values calculated by multi-objective optimization and integrated design and control approach.

Decision Variable	IMR		
	OD	IDC	
		*J_COST_*	*J_MPC_*
Steamer HEX area (m^2^)	0.04	0.30	0.25
HEX area (m^2^)	0.23	0.45	0.30
Membrane diameter (m)	0.013	0.017	0.014
Reactor outer diameter (m)	0.036	0.093	0.070
Molten salt outer diameter (m)	0.078	0.139	0.099
Reactor length (m)	0.157	0.583	0.478
Condenser HEX area (m^2^)	0.04	0.38	0.49
Water inlet flow (×10^−5^ m^3^/s)	1.90	1.76	1.77
Methane inlet flow (×10^−5^ m^3^/s)	0.37	0.43	0.43
Splitter 1 ratio (-)	0.60	0.86	0.84

**Table 4:** Decision variables for the CRM and CRMRM flowsheet configurations. Comparison between multi-objective optimization results and integrated design and control approach.

Decision Variable	CRM			CRMRM		
	OD	IDC		OD	IDC	
		*J_COST_*	*J_MPC_*		*J_COST_*	*J_MPC_*
Steamer HEX area (m^2^)	0.15	0.38	0.39	0.46	0.43	0.43
HEX area (m^2^)	0.24	0.46	0.42	0.33	0.36	0.35
Condenser HEX area (m^2^)	0.05	0.12	0.04	0.06	0.08	0.03
Water inlet flow (×10^−5^ m^3^/s)	6.70	6.68	6.79	200.20	238.56	239.8
Methane inlet flow (×10^−5^ m^3^/s)	1.10	1.70	1.75	39.00	45.42	56.39
Reactor 1 Ri (m)	0.030	0.063	0.055	0.028	0.089	0.088
Reactor 1 Ro (m)	0.126	0.159	0.160	0.369	0.212	0.209
Reactor 1 L (m)	0.144	0.696	0.644	0.661	1.341	1.346
Separator 1 Ri (m)	0.006	0.024	0.012	0.046	0.004	0.009
Separator 1 Ro (m)	0.121	0.188	0.174	0.085	0.127	0.130
Separator 1 L (m)	0.267	0.416	0.547	0.179	0.600	0.609
Reactor 2 Ri (m)	-	-	-	0.096	0.078	0.070
Reactor 2 Ro (m)	-	-	-	0.297	0.373	0.374
Reactor 2 L (m)	-	-	-	2.188	2.710	2.729
Separator 2 Ri (m)	-	-	-	0.037	0.055	0.059
Separator 2 Ro (m)	-	-	-	0.128	0.066	0.066
Separator 2 L (m)	-	-	-	0.323	0.653	0.649
Splitter 1 ratio (-)	0.59	0.87	0.87	0.80	0.70	0.69
Splitter 2 ratio (-)	0.42	0.17	0.16	0.41	0.91	0.90
Splitter 3 ratio (-)	-	-	-	0.32	0.49	0.50
Splitter 4 ratio (-)	-	-	-	0.08	0.77	0.78
Splitter 5 ratio (-)	-	-	-	0.81	0.58	0.58

**Table 5:** Normalized values of each term of the objective function at the optimal point for the three alternative flowsheets, alternative procedures (IDC and OD) and alternative indices (*J_COST_* and *J_MPC_*).

Variable	IMR			CRM			CRMRM		
	OD	IDC		OD	IDC		OD	IDC	
	*J*_1_ + *J*_2_	*J*_1_ + *J*_2_ + *J_COST_*	*J*_1_ + *J*_2_ + *J_MPC_*	*J*_1_ + *J*_2_	*J*_1_ + *J*_2_ + *J_COST_*	*J*_1_ + *J*_2_ + *J_MPC_*	*J*_1_ + *J*_2_	*J*_1_ + *J*_2_ + *J_COST_*	*J*_1_ + *J*_2_ + *J_MPC_*
Equipment cost	1	1.07	1.09	1.03	1.14	1.25	2.96	3.17	3.23
Operational cost	1	1.21	1.23	3.10	5.40	5.44	10.64	12.09	12.46
Dynamic cost	1	0.83	0.84	0.63	0.49	0.55	0.63	0.47	0.45
